# Editorial: The Evolving Chromatin and Transcriptional Landscapes—Emerging Methods, Tools and Techniques

**DOI:** 10.3389/fcell.2021.782776

**Published:** 2021-11-01

**Authors:** Steve Bilodeau, Christoph Franz Kurat, Jean-Philippe Lambert

**Affiliations:** ^1^Centre de Recherche du CHU de Québec – Université Laval, Axe Oncologie, Québec, QC, Canada; ^2^Centre de Recherche sur le Cancer de L'Université Laval, Québec, QC, Canada; ^3^Centre de Recherche en Données Massives de L'Université Laval, Québec, QC, Canada; ^4^Département de Biologie Moléculaire, Biochimie Médicale et Pathologie, Faculté de Médecine, Université Laval, Québec, QC, Canada; ^5^Molecular Biology Division, Biomedical Center Munich, Ludwig-Maximilians-Universität, Munich, Germany; ^6^Centre de Recherche du CHU de Québec – Université Laval, Axe Endocrinologie et Néphrologie, Québec, QC, Canada; ^7^Département de Médecine Moléculaire, Faculté de Médecine, Université Laval, Québec, QC, Canada

**Keywords:** chromatin, chromatin immunoprecipitation (ChIP), transcription, epigenetics, histones

Mechanisms controlling the packaging of the genetic material into chromatin are central for normal and disease development. At the core of the chromatin structure, the DNA is wrapped around histone proteins to create nucleosomes which are constantly modified and acted upon to allow for effective regulation of transcription, DNA repair, replication and maintenance of the cellular state. Accordingly, in recent years, multiple chromatin modifiers and remodelers have emerged as causal factors and promising drug targets for numerous pathologies (Hogg et al., 2020; Bhat et al., 2021). As such, an in-depth understanding of the mechanisms required for effective regulation of chromatin states during normal and disease development is essential.

The advent of effective sequencing technologies has enabled rapid progress in our understanding of chromatin biology. For example, the original article by Bae and Lesch made use of the chromatin immunoprecipitation coupled to sequencing (ChIP-seq) technique to highlight bimodal patterns of H3K4me1 at active promoters flanked by H3K4me3. Interestingly, a unimodal pattern was found to coincides with H3K4me3 and H3K27me3 at poised promoters. Furthermore, emerging sequencing techniques were the basis of the thought-provoking opinion article of Khelifi and Hussein on the roles of RNA directed interactions on genome organization. The authors postulate that two distinct functional groups of long non-coding RNA (lncRNA) respectively operate locally on the structure of chromatin itself and promote long-range chromatin interactions and bridging events.

This Frontiers Research Topic reports significant progresses toward the systematic deployment of complementary approaches to sequencing techniques. One example is the development of degenerated methylated lysine-oriented peptide libraries (Kme-OPL), which enables the specificity of Kme reader modules to be defined. In a research article, Kupai et al. describe the development of Kme-OPL and its use for the characterization of Kme reader modules to reveal the specificity or promiscuity of Kme reader modules. Similarly, Janna et al. details the biochemical and structural studies of the crosstalk between PTMs which enable a molecular understanding of the positive impact of histone H2B ubiquitylation on the methylation of H3K79 and H3K4. This is furthered by Scott and Campos who discuss the numerous tools to characterize histone H3 and its partners. One such tool, proximity dependent biotinylation is highlighted by Ummethum and Hamperl.

In this Frontiers Research Topic, three detailed protocols by Aziz Khan et al.; Galloy et al.; and Robu et al. promote the effective characterization of chromatin and its effectors. In their step-by-step protocol, Aziz Khan et al. describe how to isolate large amounts of nucleosomes from mammalian cells for downstream characterization. Galloy et al. focuses on chromatin remodelers and provided two distinct protocols to permit large-scale purification of chromatin remodeling complexes and the use of an anchor-away system in human cells. Lastly, Robu et al. reports step-by-step protocols to study proteins involved in nucleotide excision repair (NER) localization at DNA lesions. These methods all contribute to the characterization of the dynamic nature of the interplays shaping the chromatin environment.

Further, the need for effective model systems to study chromatin was also highlighted in this Frontier Research Topic. In a brief research report, Karányi et al. revisited the roles of H3K56ac during meiotic recombination. Working in the atypical SK1 *Saccharomyces cerevisiae* strain, a strain well-adapted to synchronous sporulation (Borner and Cha, 2015), the authors employed classical yeast genetics in combination with ChIP-seq to reveal the requirement for H3K56ac to produce normal levels of double strand breaks in recombination hotspot regions. In a review article, Wahab et al. highlight the *Tetrahymena thermophila* model, its unique biology and its use to study Kac-dependent processes. Historically *Tetrahymena* has enabled the identification of the first lysine acetyltransferase (Brownell et al., 1996). The authors propose that it is perfectly suited to uncover novel mechanisms impacting chromatin structures and functions when coupled to modern techniques.

While sequencing-based methods remain the dominant approach to study chromatin biology, exciting new tools and techniques are emerging to complement them. Together, these approaches will allow for a more detailed understanding of chromatin biology and transcriptional regulation. We believe that this Frontiers Research Topic will support this endeavor.

## Author Contributions

All authors listed have made a substantial, direct and intellectual contribution to the work, and approved it for publication.

## Funding

Research in the Bilodeau Laboratory was funded by funds from the Canada Research Chair in Transcriptional Genomics [Grant #950-228321]; a Discovery Grant from the Natural Sciences and Engineering Research Council of Canada (RGPIN-2019-06490), a Project Grant from the Canadian Institutes of Health Research (PJT-451568) and an operating grant from the Cancer Research Society and Genome Quebec (840369). Research in the Kurat Laboratory was funded by the Deutsche Forschungsgemeinschaft (DFG, German Research Foundation; Project-ID 213249687–SFB 1064). Research in the Lambert Laboratory was funded by a Discovery Grant from the Natural Sciences and Engineering Research Council of Canada (RGPIN-2017-06124), a Project Grant from the Canadian Institutes of Health Research (PJT-168969) and an operating Grant from the Cancer Research Society and Genome Quebec (25123).

## Conflict of Interest

The authors declare that the research was conducted in the absence of any commercial or financial relationships that could be construed as a potential conflict of interest.

## Publisher's Note

All claims expressed in this article are solely those of the authors and do not necessarily represent those of their affiliated organizations, or those of the publisher, the editors and the reviewers. Any product that may be evaluated in this article, or claim that may be made by its manufacturer, is not guaranteed or endorsed by the publisher.
